# Likelihood ratio of computed tomography characteristics for diagnosis of malignancy in adrenal incidentaloma: systematic review and meta-analysis

**DOI:** 10.1186/s40200-016-0224-z

**Published:** 2016-04-21

**Authors:** Fatemeh Alsadat Sabet, Reza Majdzadeh, Babak Mostafazadeh Davani, Kazem Heidari, Akbar Soltani

**Affiliations:** 1 School of Medicine and Public Health, Evidence based Practice Research Center, Tehran University of Medical Sciences, Tehran, Iran; 2 Center for Knowledge Translation and Exchange, Tehran University of Medical Sciences, School of Public Health, Tehran, Iran; 3 Evidence based Practice Research Center, Endocrinology and Metabolism Research Center, Tehran University of Medical Sciences, Tehran, Iran; 4 School of public health, Tehran University of Medical Sciences, Tehran, Iran

**Keywords:** Adrenal incidentaloma, Malignancy, Systematic review, Likelihood ratio, HU Density

## Abstract

**Purpose:**

To propose an evidence based diagnostic algorithm using mass characteristics to determine malignancy in patients with adrenal incidentaloma by CTscan.

**Methods:**

A systematic review in Medline, Scopus, relevant reference books and desk searching was performed up to January 2016 with relevant reference checking. The summery estimates of sensitivity, specificity, positive and negative likelihood ratio of different characteristics were calculated in two groups of the articles investigating the cases without previous malignancy and the articles investigating the oncologic cases.

**Results:**

Thirty six articles were included in this study. In the first group with no history of malignancy a positive and negative LR of 3.1 and 0.13 in 4 cm threshold and positive and negative LR of 2.85 and 0 in 10HU density were found. In the second group with history of malignancy positive and negative LR of 2.3 and 0.27 in 3 cm threshold and positive and negative LR of 3.6 and 0.08 in 20HU density were resulted.

**Conclusion:**

The results retrieved in this study considering the limitations show that adrenal incidentaloma with a size less than 4 cm or a mass larger than 4 cm with density less than 10HU in the first group can be managed with imaging follow up. For masses larger than 4 cm with density more than 10HU another diagnostic procedure should be performed. In the second group an adrenal mass larger than 3 cm or less than 3 cm with density more than 20HU should go under operation. But masses smaller than 3 cm with less than 20HU density can be followed by imaging.

## Background

Adrenal incidentaloma, a clinically silent adrenal mass, detected inadvertently during diagnostic tests or treatment for causes other than adrenal disease, was first described more than 20 years ago [1]. During recent years, widespread application of noninvasive and sensitive imaging techniques has led to an increased detection of incidentalomas [2–5]. These adrenal masses occur in 0.35–9 % of all abdominal computerized tomography (CT) scans [2, 3, 6–8] that increases to as much as 10 % in the elderly [1] peaking in fifth and seventh decades [5]. In autopsies or cases of prior malignancy the prevalence of previously undetected adrenal tumors increases again [3, 4, 8–10].

36–94 % of these masses are benign cortical adenomas [3, 4, 9, 11–15], and even in patients with a known carcinoma of any histology only 19–75 % of the masses are metastatic [7, 8, 15–18]. However, because of high mortality rate of malignant lesions with less than 50 percent 5-year overall survival for adrenal cortical carcinoma, it is needed to rule out these lesions [7, 11]. The prevalence of adrenal carcinoma reaches to 3–17 per million in general population, however, adrenal incidentaloma has a chance of 1.2–12 % to be malignant [7, 8, 19, 20].

The first step in the evaluation of an adrenal mass is to classify it as a hormonally active or a nonfunctional mass [21, 22]. In the second step the mass should be verified for the risk of malignancy. Typical characteristics of benign adenomas on CT include smooth contour, sharp margin and small size, while typical features of carcinoma are heterogeneity, tumor calcification and large size [23, 24]. The problem is that significant overlap with malignant lesions limits the usefulness of size as a criterion [25]. The nature of incidentally found adrenal masses is more questionable when the size is 3–6 cm [7, 26–28]. It is important to keep in mind that with present strategies, the diagnosis process is not cost-effective with more than necessary rate of operation and the need for a better guideline is repeated in the literature [24, 29].

The aim of this study was to answer what are the Likelihood Ratios (LRs) of different characteristics of adrenal masses in CT scan as the method of choice to diagnose malignancy in patients with adrenal incidentaloma. Subsequently we purposed to suggest an evidence based flowchart for evaluation of malignancy of the adrenal mass by CT scan helping us to choose the best time for operation.

## Methods

### Literature search and study selection

In this study we searched Medline and Scopus databases from 1970 to January 2016 by structured search strategy including both text word and Medical Subject Heading (MeSH) term of any of the following headings: (adrenal incidentaloma OR adrenal mass) AND diagnostic search strategy [30]. Our complete search strategy in Medline database was:

(("adrenal incidentaloma"[Text Word] OR "adrenal incidentaloma"[Mesh] OR "adrenal mass"[Text Word] OR "adrenal tumor"[Text Word]) AND (("physical examination"[MeSH Terms] OR physical examination[Text Word]) OR ("medical history taking"[MeSH Terms] OR medical history taking[Text Word]) OR ("professional competence"[MeSH Terms] OR professional competence[Text Word]) OR ("sensitivity"[MeSH Terms] OR sensitivity[Text Word] OR specificity [MeSH Terms] OR specificity[Text Word]) OR ("reproducibility of results"[MeSH Terms] OR reproducibility of results[Text Word]) OR ("observer variation"[MeSH Terms] OR observer variation[Text Word]) OR ("routine diagnostic tests"[Text Word] OR "diagnostic tests, routine"[MeSH Terms] OR diagnostic tests[Text Word]) OR ("decision support techniques"[MeSH Terms] OR decision support techniques[Text Word]) OR ("bayes theorem"[MeSH Terms] OR Bayes theorem[Text Word]) OR ("predictive value of tests"[MeSH Terms] OR predictive value of tests[Text Word]) OR ("palpation"[MeSH Terms] OR palpation[Text Word]) OR ("percussion"[MeSH Terms] OR percussion[Text Word]) OR ("diagnosis"[Subheading] OR "diagnosis, differential"[MeSH Terms] OR differential diagnosis[Text Word]) OR ("diagnostic errors"[MeSH Terms] OR diagnostic errors[Text Word])) AND ((Humans[Mesh Terms]) AND (English[lang])))

We used the same strategy for searching articles in Scopus database. Our search covered references of the related chapters of relevant textbooks [31–34] as well as desk searching. To be sure about the acceptable coverage of the study, the references of the selected articles were also reviewed and the relative articles among the references were chosen to be appraised. This method was continued until no more new articles were found.

Two independent reviewers (F.S. and B.M.) chose the potentially relevant articles retrieved by the search based on the previously set inclusion and exclusion criteria. According to the inclusion criteria the a) original articles b) published after 1970 c) which were in English d) discussed CT scan as the diagnostic test in which e) a gold standard test (operation, biopsy, FNA or follow up for more than 6 months) was performed for final diagnosis were selected. The other inclusion criteria of relevant full-texts were f) the presence of full explanation of imaging procedure that follows standard method of CT scanning and g) the presence of clearly described criteria for index test with accepted thresholds. Then the articles found a) overlapped with the others, b) the articles without any case of malignancy or c) without any case of benign mass or d) “case report” or “case series” articles with less than 15 subjects were excluded from the study. The reviewers reached agreement on all challenging studies for inclusion by face-to-face discussion.

### Quality assessment and data extraction

The included articles underwent appraisal by two reviewers independently for quality based on Quality Assessment of Diagnostic Accuracy Studies standard checklist (QUADAS) [35] and consensus was reached through discussion for different opinions. QUADAS tool is an evidence-based checklist with 14 questions, developed for evaluation of diagnostic accuracy studies. In case of any disagreement remained after discussion we referred to the third reviewer opinion (A. S.). The extracted data from each study included date of publication, place and time in which the study was conducted, number of subjects, the range and mean of the age of the subjects, special characteristics of subjects, the reference test and the type of study whether it is retrospective or prospective. For imaging features mass size, appearance characteristics of the mass like heterogeneity, irregularity, smoothness of margin and calcification in non-contrast CT scan as well as Hounsfield Unit (HU) on CT scan were extracted and enhancement characteristics of the mass were specified.

The studies were classified into two categories: the studies including true adrenal incidentalomas without any history of malignancy and the studies based on the subjects who had a prior history of neoplasm. The studies that did not separate these two groups of subjects clearly or surgical series that did not exclude known extra-adrenal malignant cases were included in second category to make sure that we have a collection of true adrenal incidentalomas in the first one. Available data on true positive (TP), false positive (FP), false negative (FN) and true negative (TN) were extracted to have the test-disease 2 × 2 table. If sensitivity and specificity were reported in the article the raw data were calculated based on total number of subjects.

### Statistical analysis

The sensitivity and specificity of a) size of the mass, b) different appearance characteristics of the mass in CT scan, c) CT scan density of the mass (based on HU) and d) enhancement characteristics in both groups of the patients with and without prior history of malignancy were calculated for each article with a 95 % confidence interval. The co-sensitivity and co-specificity (pooled sensitivity and specificity of the cases calculated by “midas” command) as well as pooled positive or negative likelihood ratios were reported. Positive LR equals to sensitivity divided by one minus specificity while negative LR equals to one minus sensitivity divided by specificity. The articles were considered homogenous for meta-analysis when the I^2^ was less than 50 considering its 95 % confidence interval reported. The co-sensitivity, co-specificity and positive and negative likelihood ratio for diagnosing malignant masses of the adrenal gland were calculated using random effect model of “midas” command in “STATA” software [36, 37]. The adequate number of data for “midas” command to analyze was 4 articles so we only pooled data of at least 4 article categories.

## Result

### Studies

**Fig. 1:**
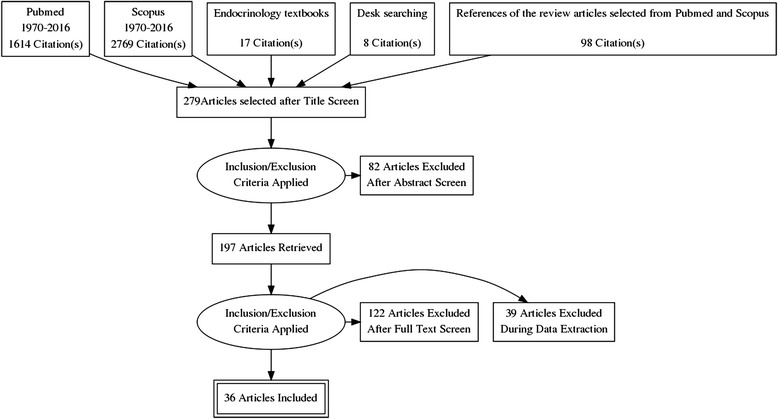
Article selection diagram

A total of 1614 studies in Medline and 2769 studies in Scopus were identified. 17 studies were found relevant to this study among references of text books of endocrinology and 8 studies were found in desk searching for diagnosis of malignancy of adrenal incidentaloma. We also found 98 references of review articles relevant to our study. The selection process was conducted based on the previously mentioned inclusion and exclusion criteria (Fig. 1) Finally 36 full texts of the articles were selected. (Table 1) The articles a) without acceptable quality using QUADAS scoring tool including the ones with inacceptable reference standards, (n:24), b) with number of subjects less than 15 (n:6), c) the studies with objectives irrelevant to our study or the imaging tool other than CT scan (n:26), d) the ones with inappropriate data reporting that did not let us to extract 2 × 2 table elements (n:39), e) the studies without any case of malignant or benign mass (n:5), f) review articles (n:59), g) the one that the subjects overlapped other studies and h) an article without appropriate method for subject selection were excluded in this level. We found two articles by Szolar in 1997 and 1998 [38, 39] that seemed overlapped but they are both included in the study extracting non-identical data from both because we did not receive any answer from the author asking about the potential overlap between subjects of studies.

**Table 1:** Details of articles included in this study using CT scan as the imaging procedure

Author	Publication date	Country, (State), period of conduction of the study	Number of subjects	Age: Mean, (range)	Description of the mass	Gold standard	QUADAS score (out of 14)	Type
Birsen [61]	2014	USA (Ohio), 2000–2012	157		Non functional	Operation, follow up	13	retrospective
Reginelli [62]	2014	Italy, 2011–2013	35	(25–89)	Non functional	Operation, follow up	11	retrospective
Allan[63]	2013	USA (Florida), 2006–2010	49	51	Functional	Operation	12	prospective
Henning [64]	2009	Sweden (Uppsala), 2001–2003	38	67.5, (45–81) 60, (24–77)	Non functional	Operation, follow up	13	retrospective and prospective
Bhargav [65]	2008	India, 1991–2005	59	41, (13–58)	Functional	Operation, FNA	10	retrospective
Vilar [66]	2008	Brazil, 2000–2007	52	50.3, (25–62)	Non functional	Operation, FNA, follow up	12	retrospective
Park [67]	2007	Korea, 2001–2005	45	46.4, (16–72)	Functional, Non functional, Metastasis	Operation	13	retrospective
Blake [68]	2006	USA, (Massachusetts), 2000–2002	99	66, (37–86)	Non functional, Metastasis, excluded Pheo, Cyst, myelolipoma	Follow up, operation, FNA	13	retrospective
Meyer [69]	2006	Germany (Hannover), 1987–2001	52	56.4	Non functional	Operation	12	retrospective
Hamrahian [53]	2005	USA, (Ohio), 1997–2002	297	54, (42–66)	Functional, Non functional, Metastasis	operation	13	retrospective
Frilling [70]	2004	Germany (Essen), 1995–2003	42	58, (33–79)	<6 cm, Metastasis	Operation, FNA,	11	prospective
Gufler [54]	2004	Germany (Siessen), 3 years	56	56.2, (34–80)	metastasis	Follow up, operation, biopsy	11	retrospective
Bulow [57]	2002	Sweden (33 Swedish hospitals), 1996–2001	381 (85 operated)	64, (18–84)	Functional, Non functional, Metastasis	operation	10	prospective
Mantero [71]	2000	Italy, 1980–1995	1004 (380 operated)	58, (15–86)	Non functional	Operation,	11	retrospective
Bergstrom [72]	2000	Sweden (Uppsala)	15	(42–78)	>1 cm Functional, Non functional	Operation, biopsy	11	prospective
Sworczak [73]	2000	Poland, 1994–1999	57	54.7, (34–79)	Non functional	Operation	12	prospective
Tutuncu [74]	1999	Turkey, 1985–1995	33 (17)		Functional, Non functional	Follow up, operation, biopsy	10	retrospective
Szolar [38]	1998	Austria	122	64, (12–87)	Functional, Non functional, Metastasis	Follow up, operation, biopsy	12	prospective
Kasperlik-Zaluska [75]	1997	Poland, 1987–1997	208	52, (14–76)	Non functional,	Operation(>4 cm), follow up	12	prospective
Boland [76]	1997	USA, (Massachusetts), 1995–1996	44	63, (21–88)	Metastasis, Non functional	Follow up, operation, biopsy	12	prospective
Szolar [39]	1997	Austria	72	60, (12–83)	Functional, Non functional, Metastasis	Follow up, operation, biopsy	12	prospective
Angeli [77]	1997	Italy, 1980–1995	887 (316)	56, (15–89)	Non functional,	operation	11	retrospective
Mc Nicholas [51]	1995	USA, (Massachusetts)	37	63, (19–81)	Metastasis	Biopsy, Follow up	13	prospective
Miyake [78]	1994	Japan	34		Non functional, Metastasis	Operation, follow up	12	prospective
Singer [79]	1994	USA, (Ohio), 1988–1991	21	66, (54–75)	Functional, Non functional, Metastasis	Operation, FNA	11	retrospective
van Erkel [80]	1994	Netherland, 1985–1991	37	(21–77)	Non functional	Operation, follow up	11	prospective
Candel [81]	1993	USA, (Illinois), 1985–1991	39	67, (29–80)	Metastasis	FNA	12	retrospective
Semelka [82]	1993	North Carolina (USA)	30	66.8, (21–82)	Not mentioned	Operation, FNA, follow up	11	prospective
Lee [83]	1991	USA, (Massachusetts), 1988	55	52, (30–75)	>1 cm,> − 50HU Metastasis, Non functional	Follow up, operation, biopsy	12	retrospective
Herrera [84]	1991	USA, (Minnesota), 1985–1989	342	62, (2–86)	Non functional	Operation, FNA, follow up	12	retrospective
Yamakita [85]	1990	Japan, 1964–1988 (1980–1988)	379	(38–65)	Non functional	Operation	11	retrospective
Lopez [86]	1990	Chile, 1984–1988	18	(21–80)	>6 cm, Functional, Non functional, Metastasis	operation	12	retrospective
Hubbard [55]	1989	USA, (Tennessee), 1978–1987	28	(22–74)	Non functional	Operation, FNA, follow up	12	retrospective
Paivansalo [40]	1988	Finland, 1980–1986	75	58, (2–83)	Functional, Non functional, Metastasis	Operation, follow up	11	prospective
Katz [41]	1985	Texas, 1977–1983	16	(35–76)	Metastasis	FNA	11	prospective
Hussain [42]	1984	USA, (Massachusetts), 1979–1983	43	38, (7–75)	Functional, Non functional, Metastasis	Biopsy, surgery	11	prospective

### Pooling of data and meta-analysis

Data were extracted in four main categories from the articles. These groups include size, appearance in CT scan, density of the mass based on HU and enhancement characteristics of the mass.

### Size

**Fig. 2:**
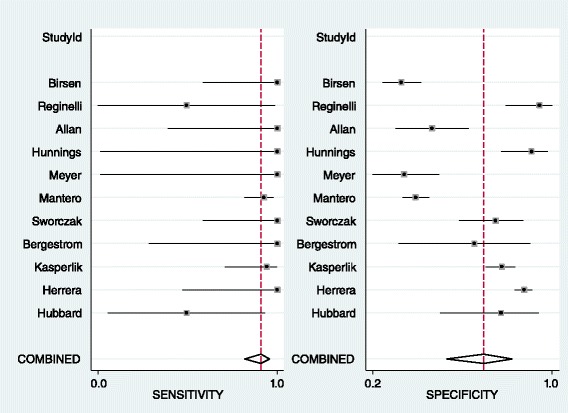
Forest plot of 4 cm adrenal mass as the best cut-off in patients with adrenal incidentaloma without history of malignancy

**Fig. 3:**
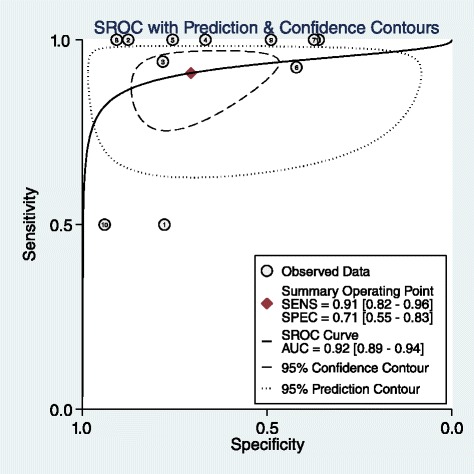
SROC of 4 cm adrenal mass as the best cut-off in patients with adrenal incidentaloma without prior history of malignancy

Results of the pooled estimate of statistical measures of the test based on different size cut-offs of the adrenal mass in the first group of patients without extra-adrenal malignancy history shows the articles in all the size cut-off groups are heterogeneous except for the 4 cm cut-off in which because of wide range of I^2^ the result is considered non homogenous as well (Table 2). As it is predicted, sensitivity for detection of malignant masses decreases with progression in size while specificity has an increasing course which makes an obvious change in 4 cm cut-off. The forest plot and Summary Roc Curve (SROC) of articles in 4 cm size category as the best cut-off for malignancy detection based on previous studies [40–46] show the articles are heterogeneous but have appropriate test accuracy measures with an area under the curve (AUC) of 0.92 (Figs. 2 and 3). No publication bias was detected for this group of articles. (Begg’s test *p*-value = 0.06 for sensitivity and 0.64 for specificity)

**Table 2:** LR for Size. Pooled estimate of sensitivity, specificity, positive and negative LR based on different size cut-offs of the adrenal mass in patients without prior history of malignancy

Cut-off	No of studies	Co-sensitivity (95 % CI)	Co-specificity (95 % CI)	I^2^ (95%CI)	Pooled positive LR	Pooled negative LR
3 cm	9	0.91, (0.83–0.95)	0.44, (0.28–0.62)	77, (50–100)	1.6, (1.2–2.2)	0.21, (0.10–0.42)
4 cm	11	0.91, (0.82–0.96)	071, (0.55–0.83)	77, (51–100)	3.1, (2–4.9)	0.13, (0.06–0.25)
5 cm	8	0.78, (0.67–0.87)	0.82, (0.65–0.91)	69 (32–100)	4.3, (2.1–8.9)	0.26, (0.16–0.44)
6 cm	11	0.74, (0.63–0.82)	0.85, (0.69–0.94)	94 (89–99)	5.0, (2.4–10.8)	0.31, (0.22–0.43)

**Fig. 4:**
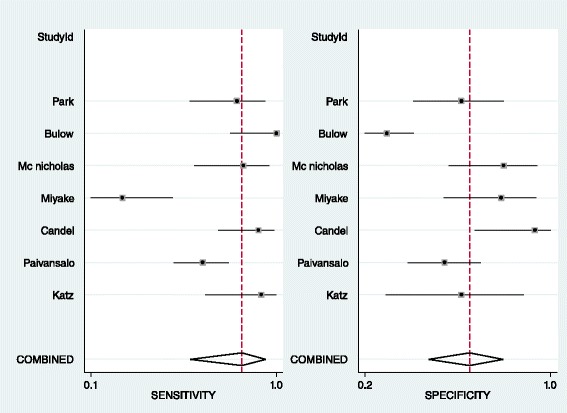
Forest plot of 3 cm adrenal mass as the best cut-off in patients with adrenal incidentaloma with history of malignancy

**Fig. 5:**
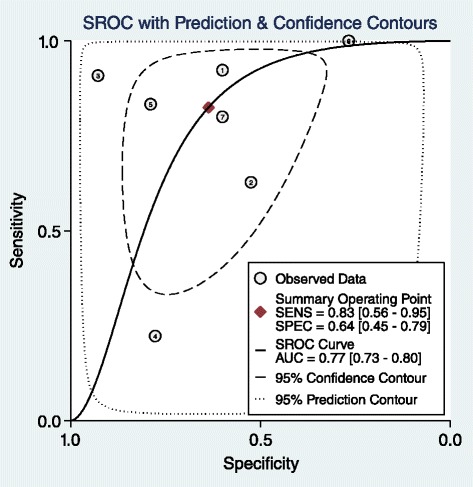
SROC of 3 cm adrenal mass as the best cut-off in patients with adrenal incidentaloma with prior history of malignancy

In patients with prior history of malignancy, the results of pooled estimates in different size cut-offs show a decrease in sensitivity with an increase in specificity in progression of size as it was predicted. All size category articles are heterogeneous regarding high I^2^ (Table 3). Based on previous studies the best size cut-off in this category for detection of malignancy is 3 cm [3, 44] with a distinct decrease in sensitivity and increase in specificity after it in this study. Forest plot shows heterogeneous articles and SROC shows an AUC of 0.77 in this size category (Figs. 4 and 5). No publication bias was detected for this group of articles. (Begg’s test *p*-value = 0.07 for sensitivity and 1 for specificity)

**Table 3:** LR for size: Pooled estimate of sensitivity, specificity, positive and negative LR based on different size cut-offs of the adrenal mass in patients with prior history of malignancy

Cut-off	No of studies	Co-sensitivity (95 % CI)	Co-specificity (95 % CI)	I^2^ (95%CI)	Pooled positive LR	Pooled negative LR
1.5 cm	1	0.93	0.16	–	1.1	0.43
2 cm	2	0.95, 1	0.41, 0.20	–	1.61, 1.25	0.12, 0
2.5 cm	3	0.79, 1, 0.84	0.84, 0.58, 0.66	–	4.93, 2.38, 2.47	0.25, 0, 0.24
3 cm	7	0.83, (0.56–0.95)	0.64, (0.45–0.79)	92, (86–99)	2.3, (1.5–3.5)	0.27, (0.1–0.74)
4 cm	6	0.56, (0.35–0.75)	0.85, (0.69–0.94)	94, (89–99)	3.8, (2.3–6.4)	0.51, (0.35–0.75)
5 cm	6	0.31, (0.14–0.57)	0.92, (0.80–0.97)	93, (86–99)	4, (1.9–8.3)	0.74, (0.56–0.98)
6 cm	6	0.32, (0.11–0.63)	0.92, (0.85–0.96)	92, (85–99)	4, (1.9–8.7)	0.74, (0.51–1.08)

### Mass appearance

The only category with proper number of studies for meta-analysis in adrenal mass CT appearance characteristics is heterogeneity in patients with prior malignancy that does not show significant positive and negative LR like other categories of mass appearance (Table 4). These results confirm low power of mass appearance for detection of malignancy.

**Table 4:** LR for mass appearance: Pooled estimate of sensitivity, specificity, positive and negative LR based on appearance characteristics of the adrenal mass in patients with or without prior history of malignancy

Mass appearance	History of malignancy	No of studies	Co-sensitivity (95 % CI)	Co-specificity (95 % CI)	I^2^ (95%CI)	Pooled positive LR	Pooled negative LR
Heterogeneity	No	3	0.79, 0.93, 0.75	0.71, 1, 0.78	–	2.72, ∞, 3.4	0.29, 0.07, 0.32
Irregularity	No	2	0.41, 0.5	0.93, 0.98	–	5.85, 0.45	0.63,
Rough margin	No	1	0.56	0.9	–	5.6	0.48
Heterogeneity	Yes	6	0.38, (0.22–0.57)	0.76, (0.66–0.84)	55, (0–100)	1.6, (0.8–3.2)	0.81, (0.57–1.16)
Rough margin	Yes	1	0.37	1	–	∞	0.63
Calcification	Yes	2	0.21, 1	0.92, 0.17	–	2.62, 1.2	0.85, 0

∞ It indicates significantly high positive LR

### Mass density in CT scan

**Fig. 6:**
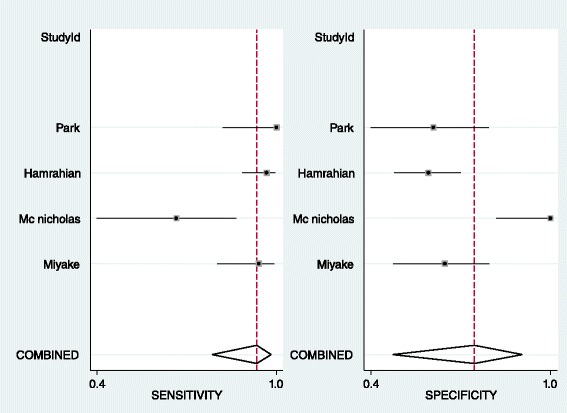
Forest plot of 20 HU adrenal mass as the best cut-off for densityin patients with adrenal incidentaloma with prior history of malignancy

**Fig. 7:**
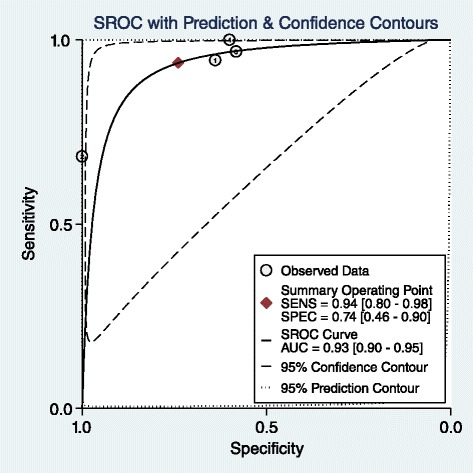
SROC of 20 HU adrenal mass as the best cut-off for density in patients with adrenal incidentaloma with prior history of malignancy

In the first group of patients without malignancy history the number of the articles in each category was not enough (were less than 4) that meta-analysis could not be performed but in second group in 10 and 20 HU thresholds the result of meta-analysis shows an increase in specificity without a marked change in sensitivity that favors 20 HU cut-off (Table 5). Previous studies propose 10 HU as the best cut-off in patients without history of malignancy [45, 47–50] and 20 HU as the best threshold in patients with history of malignancy [44] The articles in 20 HU mass density are heterogeneous based on forest plot resulted in this study (Fig. 6). SROC with an AUC of 0.93 shows an appropriate test accuracy measure (Fig. 7). No publication bias was detected for this group of articles. (Begg’s test *p*-value = 0.30 for sensitivity and 1 for specificity)

**Table 5:** LR for mass density. Pooled estimate of sensitivity, specificity, positive and negative LR based on density of the adrenal mass in patients with or without prior history of malignancy

Mass density cut-off	History of malignancy	No of studies	Co-sensitivity (95 % CI)	Co-specificity (95 % CI)	I^2^ (95%CI)	Pooled positive LR	Pooled negative LR
10 HU	No	1	1	0.65	–	2.85	0
16 HU	No	1	0.95	1	–	∞	0.05
20 HU	No	1	1	0.81	–	5.26	0
0 HU	Yes	3	1,1,1	0.47, 0.72, 0.33	–	1.88, 3.57, 1.49	0,0,0
10 HU	Yes	7	0.96, (0.86–0.99)	0.52, (0.39–0.64)	77, (50–100)	2, (1.5–2.6)	0.08, (0.02–0.3)
11 HU	Yes	1	1	0.55	–	2.22	0
13 HU	Yes	1	1	1	–	∞	0
15 HU	Yes	1	1	0.64	–	2.77	0
18 HU	Yes	1	0.95	0.82	–	5.27	0.06
20 HU	Yes	4	0.94, (0.80–0.98)	0.74, (0.46–0.90)	87, (73–100)	3.6, (1.6–8.3)	0.08, (0.03–0.24)
21 HU	Yes	1	0.89	1	–	∞	0.11
25 HU	Yes	1	0.95	0.72	–	3.39	0.06
35 HU	Yes	1	0.54	1	–	∞	0.46

∞ It indicates significantly high positive LR

### Mass enhancement

In 1997 Boland et al. reported sensitivity and specificity of 78 and 22 % in delayed enhancement with 28 HU threshold that reaches to 96 and 96 % with a 15 min delay and a 24 HU cut-off. [51] In the other study Szolar et al. in 1998 found a sensitivity and specificity of 97 and 100 % in 37 HU after 30 min of enhancement and 100 and 97 % in 42 HU after 30 minutes [39]. Results of other articles studying enhancement did not show enough number of articles to perform meta-analysis (Table 6).

**Table 6:** LR for mass enhancement. Pooled estimate of sensitivity, specificity, positive and negative LR based on enhancement of the adrenal mass in patients with prior history of malignancy

Mass character	History of malignancy	No of studies	Sensitivity	Specificity	Positive LR	Negative LR
RPW 10 min delay (37.5 %)	Yes	1	1	0.95	20	0
APW 10 min delay (52 %)	Yes	1	1	0.98	50	0
Contrast enhancement	Yes	1	0.74	0.83	4.35	0.31

## Discussion

The growing number of incidentally detected adrenal mass makes the diagnosis of malignant ones a challenge these days and lack of optimized diagnostic guidelines and controversies in this field represents the importance of new studies. In this study in the first group without prior history of malignancy a positive and negative LR of 3.1 and 0.13 and an area under the curve of 0.92 was found in 4 cm cut-off for detection of malignancy. The positive and negative LR are nor confirmative nor exclusive for malignancy that confirm other studies suggesting that other parameters beside size are needed for definite diagnosis [8, 45]. Although the size of the lesion is the first important known parameter to distinguish benign from malignant adrenal masses, it has a range of 3 to 6 cm for malignancy detection in different review articles because of the significant overlap between benign and malignant masses, but the most recent ones suggest 4 cm as the optimum size for operation [43–48, 52]. As it was predicted the sensitivity of detection of malignant cases decreased with size progression while the specificity increases that supports previous studies [53–55].

In the second group with an extra adrenal history of neoplasm, positive and negative LR was 2.3 and 0.27 in turn which are not confirmative or exclusive. Based on the reports of other studies, the best size threshold of masses in patients with history of malignancy is 3 cm because the prevalence of metastatic lesion increases in these studies and a metastatic lesion can be found with variable sizes as an adrenal mass [3, 44]. The process of decreasing sensitivity and increasing specificity with higher sizes as it is predicted confirm previous studies.

In accordance with other studies, the appearance of the lesion including heterogeneity, rough margins, irregularity and calcification does not show a potent positive and negative LR. Although usually the malignant masses are more heterogeneous with irregular margins, some benign masses can appear irregular as well [45, 46]. The low strength likelihood ratios in the second group shows that appearance of the mass cannot be so helpful to differentiate malignant lesions in patients with extra-adrenal malignancy because of higher prevalence of metastatic lesions in this group which can be similar in appearance with adenoma, which shows similarity with previous findings [18].

In this study in the first group without history of neoplasm in 10 HU cut-off for density a positive and negative LR of 2.85 and 0 was found. Hounsfield unit of the mass is considered a parameter as important as the size or even more important to distinguish malignant masses [8, 45] The density of 10 HU is reported as the best cut-off for diagnosis of malignancy according to the previous studies [45, 47–50]. In a meta-analysis in 1998 Boland et al. found a sensitivity of 71 % and a specificity of 98 % with 10 HU cut-off as the best diagnostic threshold [56]. In the second group a positive and negative LR of 3.6 and 0.08 for the density of 20 HU was the result of the present study. Based on a review on previous studies the best mass density to be chosen as treatment cut-off is 20 HU in patients with previous malignancy history [44]. Hamrahian et al. in 2005 proposed 20 HU density cut-off to perform operation in case of lower sized masses in patients with prior malignancy as well, although in that study the size threshold for operation is suggested to be 4 cm [57].

Mass washout in dynamic CT scanning is another factor that can differentiate malignancy; however just the positive and negative LR are reported in this study due to low number of articles considering this factor and wide range of techniques and cut-offs.

**Fig. 8:**
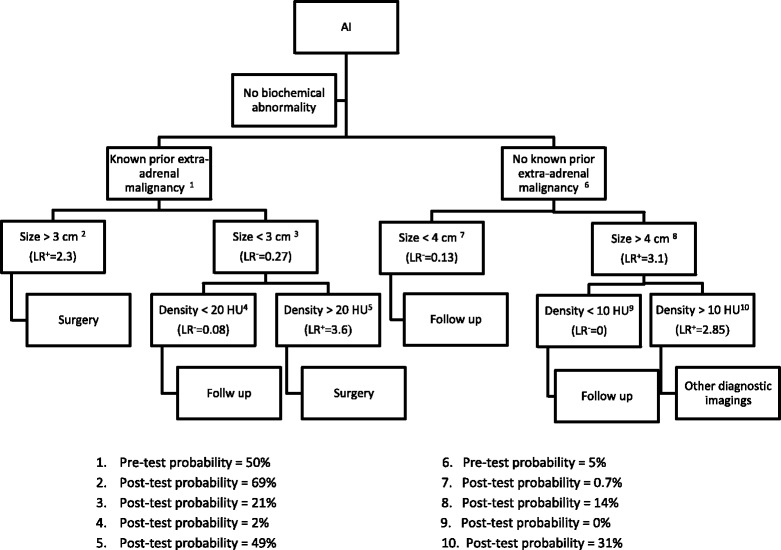
flowchart of algorithmic approach to characterization of adrenal incidentaloma

In the first group truly diagnosed with adrenal incidentaloma, with a treatment threshold of 25 % to perform operation [7], the results show a lower treatment threshold of 3 % and upper treatment threshold of 52 % for 4 cm cut-off. In previous studies prevalence of malignancy among adrenal masses including metastasis and primary adrenal tumor is reported with a range of 2.7 to 13 % in patients without extra-adrenal malignancy [8, 18, 29, 46, 47, 58]. In the studies with more restricted definition for adrenal incidentaloma a lower prevalence is reported while the reports show higher prevalence in surgical series. So the pretest probability of malignancy in true adrenal incidentalomas is assumed to be 5 % as an average in this study. The results (Fig. 8) confirms previous studies which mention that the size of the adrenal mass alone cannot be an indicator for malignancy because masses larger than 4 cm do not pass any treatment threshold [7, 26, 47]. In this condition another factor that can be diagnostic is the density of the mass in CT scan which is introduced as the best characteristic to diagnose malignancy in some studies [8, 45].

In the second group with extra adrenal neoplasm the treatment threshold is estimated 25 % [7]. In size cut-off of 3 cm as the best threshold for malignancy, there are a lower and upper treatment threshold of 8 and 43 %. The prevalence of metastasis and primary adrenal carcinoma in this group ranges from 19 to 75 % according to the literature [3, 7, 8, 15–18, 44, 59]. Although some studies indicate that the prevalence of metastasis in patients with a known neoplasm reaches 26 to 36 % [60]. Regardless of this wide range 50 % is chosen as pretest probability of malignancy in this group based on the most frequent reports.

## Conclusion

As a conclusion, an evidence based flowchart is suggested in which among the patients without history of malignancy adrenal masses smaller than 4 cm or the ones larger than 4 cm with density of less than 10 HU can be just followed up but the lesions larger than 4 cm with density more than 10 HU should be gone under additional diagnostic procedure. In patients with prior malignancy the masses larger than 3 cm or smaller than 3 cm with density more than 20 HU should be resected through surgery but the ones less than 3 cm with density less than 20 HU can be followed-up (Fig. 8).

Some limitations in this study should be considered. First, the articles are limited to English language and though the search was continued until no new article was found, there may be some studies left including conference presentation because of limitation of the search to Medline database. Second, the articles included are heterogeneous in results so the “random-effect” analysis was performed. Third, because of differences in results reported in studies in differentiation between malignant masses and non-malignant ones or adenoma and non-adenoma both were considered the same in this study to avoid several categorizations and low number of articles in each group. Forth, the studies in which functional masses and non-functional ones had not been separated were not excluded from the study. Fifth, there are reports showing that some of the adrenal metastatic lesions may not be detected within a 6 month follow up period so this diagnosing tool may not be a gold standard to detect malignancy in patients with history of neoplasm.

Although this study attempted to provide an evidence-based algorithm for approaching adrenal incidentaloma, considering its limitations, similar systematic reviews are needed to be conducted in future to collect higher number of studies. In addition, because many adrenal masses are found incidentally by other imaging methods such as MRI and Ultrasonography, assessing their ability to distinguish malignancy would be beneficial.

### Ethics approval

Not Applicable.

### Consent for publication

Not Applicable.

### Availability of data

Available on request.

## Abbreviations

AUC: area under the curve
CT: computed tomography
FN: false negative
FP: false positive
HU: Hounsfield Unit
LR: likelihood ratio
QUADAS: Quality Assessment of Diagnostic Accuracy Studies
SROC: Summary Roc Curve
TN: true negative
TP: true positive
